# Protective Effect of HMG-CoA Reductase Inhibitor Rosuvastatin on Doxorubicin-Induced Cognitive Impairment, Oxidative Stress and Neuroinflammation: Possible Role of CREB, ERK1/2, and BDNF

**DOI:** 10.1007/s11481-025-10213-6

**Published:** 2025-05-13

**Authors:** Yesim Yeni, Betul Cicek, Ahmet Hacimuftuoglu, Mustafa Ozkaraca, Burak Batuhan Lacin

**Affiliations:** 1 Faculty of Medicine, Department of Medical Pharmacology, Malatya Turgut Ozal University, Malatya, Turkey; 2 Faculty of Medicine, Department of Physiology, Erzincan Binali Yildirim University, Erzincan, Turkey; 3 Faculty of Medicine, Department of Medical Pharmacology, Ataturk University, Erzurum, Turkey; 4 Faculty of Veterinary Medicine, Department of Pathology, Sivas Cumhuriyet University, Sivas, Turkey; 5 Faculty of Veterinary Medicine, Department of Physiology, Ataturk University, Erzurum, Turkey; 6 Faculty of Medicine, Department of Pharmacology, Malatya Turgut Ozal University, Battalgazi-Malatya, 44210 Turkey

**Keywords:** Cognitive impairment, Doxorubicin, Neuroinflammation, Neuroplasticity, Rosuvastatin

## Abstract

During or after chemotherapy, cognitive impairments characterized by forgetfulness, difficulty concentrating, and depressive and anxiety-like symptoms are observed. There is limited research examining the effects of rosuvastatin (RVS), an HMG-CoA reductase inhibitor, in the context of neuroinflammation-related cognitive disruption. Here, we aimed to investigate the neuroprotective potential of RVS against doxorubicin (DOX)-induced cognitive impairments. Experimental groups were planned as control (normal saline, intraperitoneal), DOX (total cumulative dose 10 mg/kg, intraperitoneal), RVS (10 mg/kg, oral, 20 days), and RVS + DOX. Efficacy was monitored by applying a battery of behavioral assessments, as well as biochemical, genetic, histopathological, and immunohistochemical examinations. Results from Morris water maze (MWM), passive avoidance, locomotion activity, and elevated plus maze (EPM) tests showed that DOX administration caused behavioral disorders. Moreover, DOX increased the levels of inducible nitric oxide synthase (iNOS), malondialdehyde (MDA), and tumor necrosis factor-α (TNF-α), while decreasing the levels of interleukin-10 (IL-10), glutathione (GSH), superoxide dismutase, catalase (SOD), endothelial nitric oxide (eNOS), and catalase (CAT). Co-treatment with RSV significantly attenuated DOX-induced behavioral changes and oxidative stress markers. In addition, similar to the immunohistochemical results, we determined that it increased the expression levels of extracellular signal-related kinases 1/2 (ERK1/2), cyclic adenosine monophosphate response element binding protein (CREB), and brain-derived neurotrophic factor (BDNF) and restored the histopathological structure of the brain. Therefore, these results indicated that RSV has a neuroprotective effect against DOX-induced cognitive impairment by reducing neurobehavioral impairments, exerting antioxidant and anti-inflammatory effects, and modulating brain growth factors.

**Graphical Abstract:**

**Figure:**
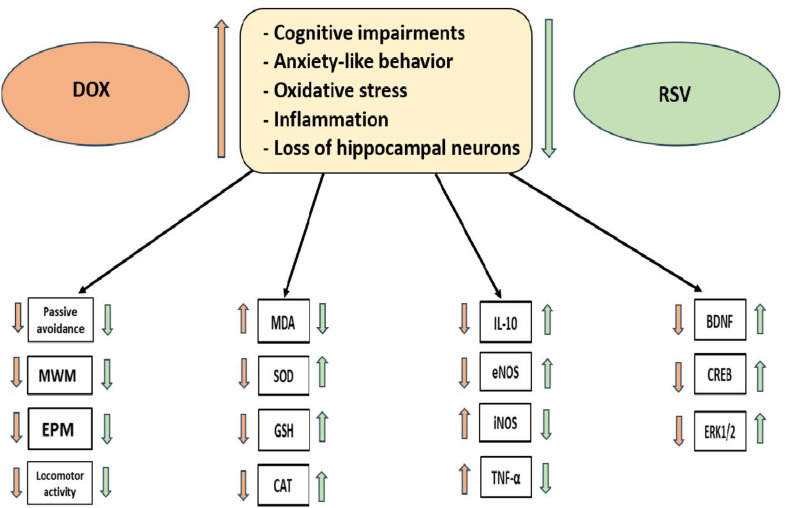
RSV ameliorated DOX-induced cognitive impairments by assessing oxidative stress, BDNF, CREB, and ERK1/2 expression. These beneficial effects induced by RSV provided strong protection against both cognitive dysfunction and anxiety-like behavior. RSV may help prevent or alleviate cognitive dysfunction in patients with various types of cancer undergoing chemotherapy and may have potential benefit in neurodegenerative diseases

## Introduction

Post-chemotherapy cognitive impairment, a clinical syndrome, is defined by chemotherapy-induced cognitive impairments like amnesia, difficulty in focusing, and multitasking. In particular, anthracycline-derived agents have been associated with more serious mind disruptions compared to other chemotherapy agents (Ali et al. 2022). Doxorubicin, belonging to the anthracycline class, is a widely used antineoplastic agent (Wu et al. 2022).

The hippocampus region of the brain, which has an important role in memory and learning, has also been reported to change during or after treatment with DOX (Ali et al. 2022; Gamal et al. 2025). DOX-induced cognitive impairment in rats has been proven by previous studies (Mounier et al. 2021; Shaker et al. 2021; Abd El-Aal et al. 2022). Moreover, DOX-related toxicity in brain tissue is much less well figured out.

Doxorubicin (DOX)-associated cognitive impairment has been attributed to increased levels of peripheral tumor necrosis factor-alpha (TNF-α), which disrupts the integrity of the blood-brain barrier and subsequently stimulates neuroinflammation via interleukin-1, interleukin-6, and nuclear factor kappa beta (Ren et al. 2019; Alsaud et al. 2023). DOX also affects other proteins such as superoxide dismutase (SOD), catalase (CAT), and glutathione (GSH) due to it causes the release of reactive oxygen species, which leads to tissue damage (Prasanna et al. 2020; (Sritharan and Sivalingam 2021).

Cyclic adenosine monophosphate response element binding protein (CREB), extracellular signal-related kinases 1/2 (ERK1/2), and brain-derived neurotrophic factor (BDNF) related to neuronal plasticity and survival, neuronal differentiation, aging, and cognitive function are important pathways in modulating cognitive formation and memory performance (Tang et al. 2020; Gao et al. 2022; Numakawa and Odaka 2021). CREB, ERK1/2, and BDNF have been identified as reliable markers to evaluate cognitive and behavioral changes associated with many neurodegenerative and psychiatric disorders. These cognitive/behavioral changes are in part attributed to the disruption of CREB, ERK1/2, and BDNF, which are included in synaptic plasticity, memory processing, and neurogenesis (Amidfar et al. 2020; Salari et al. 2024).

Recently, there is increasing evidence demonstrating the relationship between BDNF/CREB/ERK and cognitive impairment (Amidfar et al. 2020; Gao et al. 2022; Fang et al. 2024). Therefore, upregulation of the BDNF/CREB/ERK system induced by statins has been the focus of attention for the effective treatment of neurodegenerative diseases (Amidfar et al. 2020). Statins have been shown to upregulate BDNF expression and BDNF/tropomyosin receptor kinase B (TrkB)-mediated intracellular signaling, including mitogen-activated protein kinase (MAPK)/ERK, phosphoinositol 3-kinase (PI3K) /protein kinase B (Akt), and phospholipase pathways, contribute to neuronal cell survival and synaptic plasticity (Nasef et al. 2021; Numakawa and Odaka 2021). It has also been found that statins generally downregulate inducible nitric oxide synthase enzyme and neuronal nitric oxide synthase enzyme levels, leading to a decrease in overall nitric oxide production (Ekladious and El Sayed 2019).

Statins are competitive inhibitors of 3-hydroxy-3-methylglutaryl-coenzyme A (HMG-CoA) reductase and are mostly cholesterol-lowering agents. They have also been reported to have pleiotropic effects, including anti-inflammatory and anti-oxidative stress properties (Lamb 2020). Rosuvastatin (RVS), a new HMG-CoA reductase inhibitor, has shown a stronger affinity for the active site of HMG-CoA reductase compared to other statins (Wu and Chen et al. 2020). They have rapid bioavailability because they do not require hepatic metabolism to activate RVSs (Storelli et al. 2022). This rapid bioavailability may result in a fast response to DOX-induced cognitive impairment. Furthermore, RVS has been noted to have neuroprotective effects in experimental stroke patterns, including focal cerebral ischemia patterns and stroke-prone hypertensive rats (Xu and Yong et al. 2020; Murthy et al. 2023). Moreover, an in vitro study has shown that RVS protects cortical neurons from N-methyl-D-aspartate-associated excitotoxicity (Domoki et al. 2010). However, there is limited research examining the effects of RVS in the context of memory impairments associated with neuroinflammation.

This study evaluated the neuroprotective potential of RVS against DOX-induced cognitive impairment. We also purposed to research the roles of ERK1/2, CREB, and BDNF pathways that regulate cognitive impairment.

## Materials and Methods

### Drugs and Chemicals

In the present experiment, DOX (10 mg/ml Doxorubicin HCI^®^) was purchased from Koçak Farma (Turkey) to induce chemophobia in rats. RVS (Colnar^®^ 10 mg, film tablet) was purchased from Arven İlaç San ve Tic. A.Ş (Turkey). Transcriptor first strand cDNA synthesis, high pure RNA isolation, and probes master mix kit (Roche, Basel, Switzerland), SOD, interleukin-10 (IL-10), inducible nitric oxide synthase (iNOS), GSH, TNF-α, endothelial nitric oxide (eNOS), malondialdehyde (MDA), CAT, ERK1/2, BDNF, and CREB primer-probe were acquired from Thermo Fisher (Waltham, USA).

### Animals

The present experiment was confirmed by the Atatürk University Local Animal Care Board Ethics Committee (No: E.2200417004). 32 male Sprague Dawley rats, weighing 200–250 g (8–10 weeks old), provided by Atatürk University Medical Experimental Application and Research Center, were used. Rats were maintained under adjusted environmental conditions with constant humidity, a temperature of 24 ± 1⁰C, and a 12/12 hour dark/light cycle. Rats were given free reach to rodent forage pellets and water. After a one-week adaptation period, the experiment was started. All experiments were performed by the Animal Research Reporting of In Vivo Experiments (ARRIVE) guidelines.

### Experimental Design

Working groups: Four experimental groups, each consisting of eight rats, were randomly separated into groups:

**Group (1)**: Control, From the beginning of the study, rats were given intraperitoneal normal saline.

**Group (2)**: DOX, On the 1st, 8th, 15th, and 22nd days, rats were injected intraperitoneally with a total cumulative DOX dosage (10 mg/kg) of 2.5 mg/kg divided into four doses (Ali et al. 2020).

**Group (3)**: RVS, RVS was given to rats five days a week by oral gavage at a dosage of 10 mg/kg for 4 weeks (Ludka et al. 2017).

**Group (4)**: DOX + RVS, RVS was given to rats by oral gavage at a dosage of 10 mg/kg five days a week simultaneously with DOX injection (days 1, 8, 15, and 22) for four weeks.

**Fig. 1:**
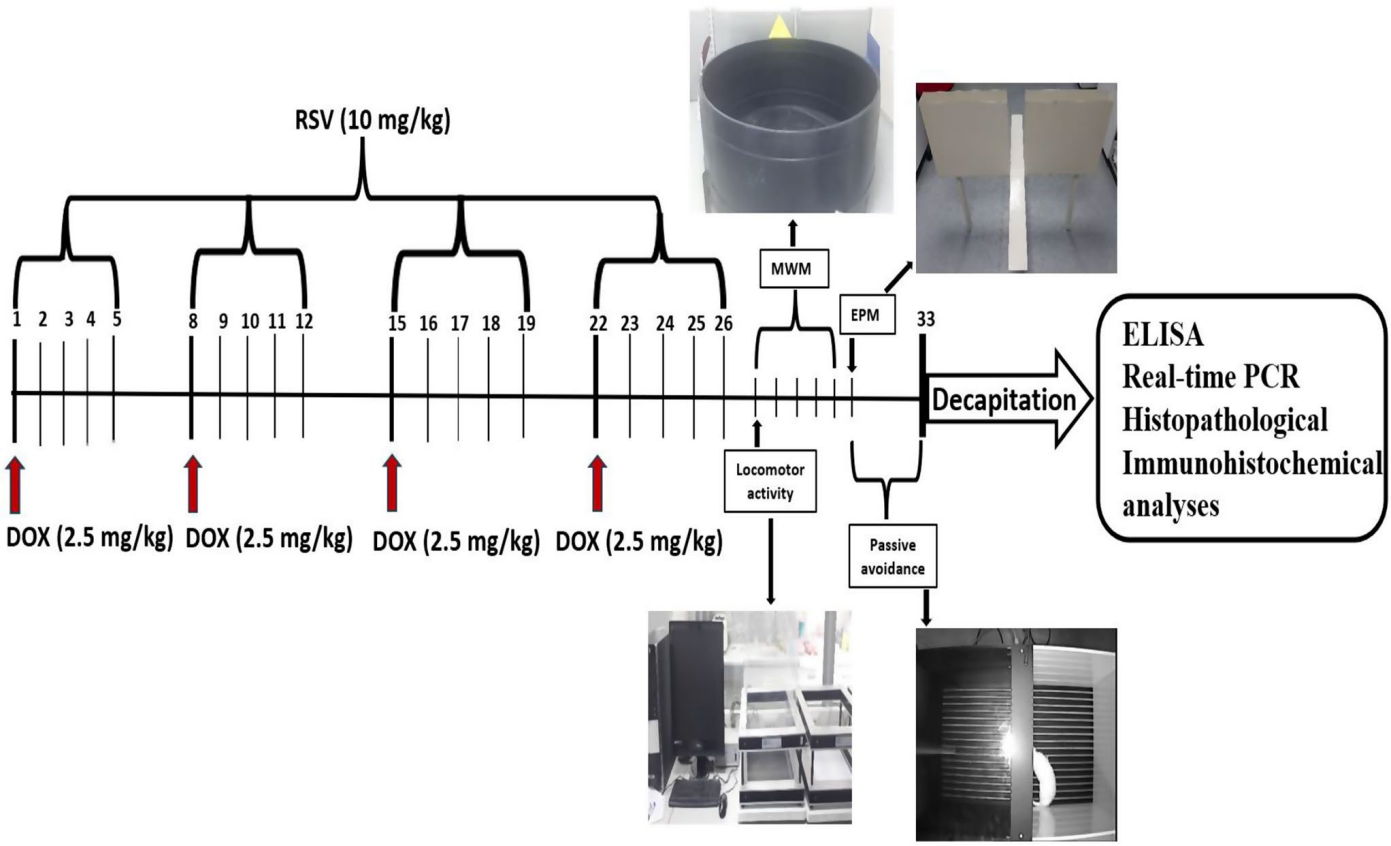
Schedule of the study’s experimental procedures

In Fig. 1, the animal treatments, behavioral tests, decapitation planning, biochemical, real-time PCR, Histopathological, and immunohistochemical analyses are made more explicit in the given timeline.

### Behavioral Tests

#### Passive Avoidance

A pace-by-pace passive-avoiding test was used to assess memory alters (Abdel-Aziz et al. 2016). Using plexiglass, the device occurred in two rooms distinct by an automatic slippery gate: the initial is lit by a white 10 W lamp, and the other is a dark room that can be programmed to deliver the required intensity of electric shock whenever a rat presses on the grid floor. Every rat was given 2 sittings: education and testing setting. In the education sitting done one week afterward the recent dosage of DOX, every rat was located in the white room. When a rat paced into the dark room and placed its 4 claws on the grating ground, the slippery gate was enclosed, and each rat was given a 1 mA electric shock for two seconds. Rats that could not pace into the dark room in 90 s were excluded from the test. The testing session was conducted 24 h afterward the education sitting, in which every rat was accommodated in the white room and their delay to insert the black room was automatically registered and took into account a pace-by-pace answer to assess their memory acquiring afterward exposure to an aversive stimulant. After recording the time to enter the dark compartment, the percentage change was calculated according to the following formula: % = (First trial − Next trial) / (First trial) × 100.

#### Morris Water Maze (MWM)

Behind the therapy, rats were tested in the MWM, an accommodated test used to appraise spatial learning and cognition (Aski et al. 2018). The pond was filled with water to a profound of 30 cm (23 ± 1^o^C), 1 cm upstairs of the platform grade. The water was then painted black with pigment paint to prevent the black platform from being visible. While this dye dyes the water black, it does not dye the rats in any way. Visual markers were attached to the walls of the pond and test chamber to canalize the rats to the position of the platform. According to the MWM protocol, the rats were allowed to swim freely in the pool for 120 s without a platform to get used to the training environment. Following the 4-day training exercise, each rat was placed face to face with the wall to reach the underwater platform and released from each of the 4 separate quadrants of the maze to explore the hidden platform. A duration spacing of 60 s was authorized for the rat to discover the platform. If the rat could not discover the platform, it was orientated to the platform and authorized to stay on the platform for 30 s to guide itself. This education sitting was reiterated for 4 sequential days. The duration to achieve the secret platform was registered and noted as a getaway lag. On the fifth day, the platform was lifted, and the memory holding of the platform position was appraised the rats’ behavior and the duration they spent in the aim quadrant were registered with a video camera placed over the pool. After recording the time spent finding the platform and in the target quadrant, the percentage change was calculated according to the following formula:

$$\% = \;({\rm{ First}}\;{\rm{trial}} - {\rm{Next}}\;{\rm{trial }})\;/\;({\rm{ First}}\;{\rm{trial }})\; \times \;100$$

$$\% \; = \;({\rm{ Last}}\;{\rm{trial}}\; - \;{\rm{First}}\;{\rm{trial}})\;/\;({\rm{First}}\;{\rm{trial}})\; \times \;100$$

#### Elevated Plus Maze (EPM) and Locomotion Activity

The anxiety-like behaviors were evaluated by utilizing the EPM (O’Hara and Co., Ltd., Japan). Each rat was placed in the middle region of the maze, facing one of the open arms. The number of entries into the open arms and the time spent were assessed with the EthoVision XT video imaging system (EthoVisionXT, Netherlands) in approximately 5 min (Yeni et al. 2024). After recording the count of entries and time spent in the open arm, the percentage change was calculated according to the following formulas:

$$\% = \;({\rm{ Open}}\;{\rm{arm}}\;{\rm{entry}}\;{\rm{count }})\;/\;({\rm{Total}}\;{\rm{entry}}\;{\rm{count}})\; \times \;100$$

$$\% \; = \;({\rm{ Open}}\;{\rm{arm}}\;{\rm{spent}}\;{\rm{time}})\;/({\rm{Total}}\;{\rm{spent}}\;{\rm{time }})\; \times \;100$$

The locomotion activity was evaluated using a four-square cage. The distance tripped and the duration spent by every rat in the centric zone were registered for 30 min by utilizing a video display system (Yeni et al. 2022).

#### Specimen Collection

After the termination of behavioral tests, rats were injected intraperitoneally with 50 mg/kg sodium thiopental (Pental^®^ IE Ulagay, Turkey). Blood samples were taken from the hearts of the anesthetized rats for biochemical analyses. They were then sacrificed via cervical decapitation. Brain tissues were collected for genetic, Histopathological, and immunohistochemical analyses. The dissected brain tissues were stored in an RNA stabilization reagent for molecular analyses. Brain tissues were fixed in 10% buffered formalin solution for immunohistochemical and histopathological examinations.

#### Determination of Oxidative Stress and Inflammatory Markers

In the present study, 5 ml blood samples were collected from rats anesthetized with intraperitoneal sodium thiopental injection (50 mg/kg) via cardiac puncture. Within 1 h after collection, it was stored on ice before centrifuging at 4000 rpm for 10 min at 4 °C (Cicek et al. 2022). Then, the supernatant was collected for the determination of biochemical indicators. IL-10, TNF-α, iNOS, eNOS, GSH, MDA, CAT, and SOD were analyzed in the obtained serum samples according to the manufacturer’s protocol of the diagnostic kits to investigate oxidative damage. The optical density of the produced colors was determined spectrophotometrically at 450 nm.

#### Gene Expression Determination

Homogenized tissue examples were first isolated by High Pure RNA isolation, followed by cDNA isolation utilizing the Transcriptor First Strand cDNA Synthesis kit to the indicated protocol. The used primers’ sequences are illustrated in Table 1. The results compared with the control group were stated as relative fold. Values were standardized to β-actin utilizing the 2^−ΔΔCT^ method (Livak and Schmittgen 2001). After the gene expression change was recorded, the percentage change was calculated according to the formula: % = (2^−ΔΔCt^ −1) × 100.

**Table 1:** Sequence list of the primers used for real-time PCR

Gene	Forward primer	Reverse primer
Β-actin	CCAACCGCGAGAAGATGA	CCAGAGGCGTACAGGGATAG
BDNF	AAGTCTGCATTACATTCCTCGA	GTTTTCTGAAAGAGGGACAGTTTAT
CREB	GACCACTGATGGACAGCAGATC	GAGGATGCCATAACAACTCCAGG
ERK1/2	TCAAGCCTTCCAACCTC	GCAGCCCACAGACCAAA

#### Histopathological Analysis

Brain tissues were fastened down in a 10% neutral formalin solution by necropsies of the rats. Tissues were gotten into paraffin blocks after routine alcohol-xylol pursuit processes. 5 µ sections taken on slides with poly-lysine were painted with hematoxylin-eosin and appraised semiquantitatively as absent (-), mild (+), moderate (++), and severe (+++) in terms of pycnotic and degenerative alterations observed in neurons (prefrontal cortex, cornu ammonia).

#### Immunohistochemical Method

Brain tissues were fastened down in a 10% neutral formalin solution by necropsies of the rats. Tissues were put into paraffin blocks after routine alcohol-xylol pursuit processes 5 μm sections taken on polylysine slides were passed the xylol-alcohol series, then washed with PBS and incubated in 3% H_2_O_2_ for 10 min to inactivate endogenous peroxidase. To disclose the antigen in the tissues, they were treated with antigen retrieval solution for 2 × 5 min at 500 watts. Tissues washed with PBS were then incubated overnight at a dilution rate of 1/150 with BDNF (Novus Bio, Catalog No. NB100-98682), Erk 1/2 (Santa Cruz, Catalog No. sc-514302), and CREB (Novus Bio, Catalog No. NB100-74393) primary antibodies. Secondarily; anti-Polyvalent, HRP (Thermo Fischer, Catalog no: TP-125-HL) was utilized as advised by the producer. 3,3′-Diaminobenzidine was utilized as the chromogen. After contrast painting with Mayer’s Hematoxylin, it was closed with entellan and investigated below a light microscope. Immunopositivity in the prefrontal cortex and cornu ammonis parts of the examination brain tissues were appraised semiquantitatively as absent (-), mild (+), moderate (++), and severe (+++) (Ekici et al. 2024).

### Statistical Analyses

The quantitative data were stated as the mean ± standard deviation. Tukey’s LSD test was used following a one-way ANOVA to assess the findings acquired in behavioral, biochemical, and molecular data. For the histopathological and immunohistochemical data obtained, the distinction between the groups was determined by the Kruskal Wallis and Mann Whitney U test. Analyzes were done using the ‘SPSS 22.0’ (IBM, USA) program. Results less than or equivalent to *p* < 0.05 were considered statistically important.

## Results

### RVS Alleviated DOX-Related Memory Degradation and Anxiety-like Behaviors

**Fig. 2:**
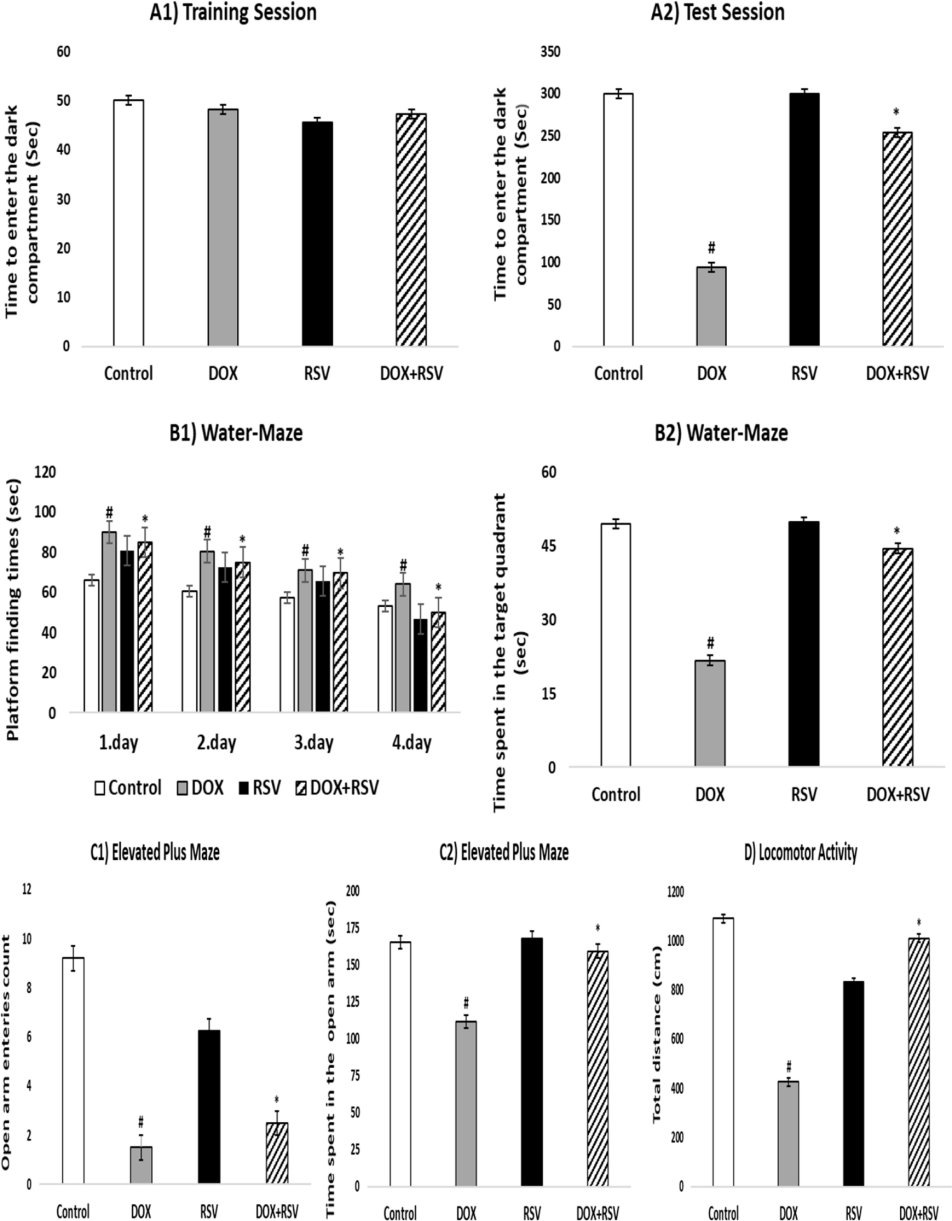
Effect of RVS therapy on DOX-related neurobehavioral alterations: (**A**) Step-through passive avoiding for the education and test session. (**B**) Assessment of Morris water maze test performance. (**C**) Assessment of elevated plus maze test performance. (**D**) Evaluation of locomotor activity test performance. Data are stated as the means ± SD (*n* = 8). #*p* < 0.001 versus the control group, *p < 0.001 versus DOX group. DOX: Doxorubicin, RVS: 10 mg/kg Rosuvastatin, DOX + RVS: Doxorubicin + 10 mg/kg Rosuvastatin

The anti-amnestic effect of RVS on DOX-related cognitive impairment was evaluated by doing the passive-avoiding test. Using this test, learning and short-term memory were assessed in rats according to our study’s training session results. Throughout the training session, there was no statistically significant difference in step latency between the treated groups, as shown in Fig. 2A1. On the other hand, in the test session, the DOX-treated group showed a significant 31% decrease in step latency compared to the control group, indicating DOX-induced impaired memory retention. Interestingly, the group simultaneously administered with RSV (85%) showed a significant difference in step latency compared to the DOX group, thus preventing the DOX-induced amnesic effect (*p* < 0.001). Furthermore, the group treated with RSV alone showed no significant difference in step latency compared to the DOX group (Fig. 2A2).

We then did the MWM analysis to further evaluate memory functions. The DOX group showed a significant delay in the time to find the platform across four training sessions, and the DOX group showed a significant increase in the time to find the platform compared to the control group. Meanwhile, the RVS and DOX + RVS groups significantly decreased the time to explore the platform compared to the DOX group (*p* < 0.001) (Fig. 2B1). During the investigation trial, the DOX (44%)-treated group was found to spend significantly less time in the target quadrant compared to the control group (*p* < 0.001). However, rats co-treated with RVS (90%) spent significantly longer in the target quadrant compared to the DOX-treated group (*p* < 0.001), indicating improved cognitive functions (Fig. 2B2). Notably, the RSV-only treated group did not show a significant difference in both platform exploration time and target quadrant time compared to the DOX group.

EPM analysis was performed to evaluate DOX-induced anxiety levels. Rats receiving DOX treatment showed anxiety-like symptoms, such as a significant decrease in the mean number of entries into the open arms by 16% (Fig. 2C1) and the time spent in the same arms by 67% (Fig. 2C2) compared to the control (*p* < 0.001). Interestingly, co-treatment with RVS reversed these changes, as confirmed by a significant increase in the mean number of entries and the time spent in the open arms by 27% and 96%, respectively, compared to the rats exposed to DOX (*p* < 0.001), confirming its anxiety-reducing effect. No significant difference was seen in the group treated with RSV alone, compared to the DOX group, in terms of both the number of entries into the open arms and the time spent in the open arms. These findings suggest that DOX treatment induces anxiety-like behaviors that can be ameliorated by RSV.

The results shown in Fig. 2D show the effect of all treated groups on locomotor activity in open-field testing. The DOX group demonstrated a significant distinctiveness compared to the control (*p* < 0.001), DOX caused a reduction in locomotor activity. On the other hand, co-administration of RVS significantly increased locomotor activity compared with the DOX group (*p* < 0.001). Also, the results correlated with the EPM. These findings suggest that locomotor activity decreases with DOX treatment and may improve with RSV support.

### RVS Effect on Oxidative Stress and Inflammatory Markers in the Serum

**Fig. 3:**
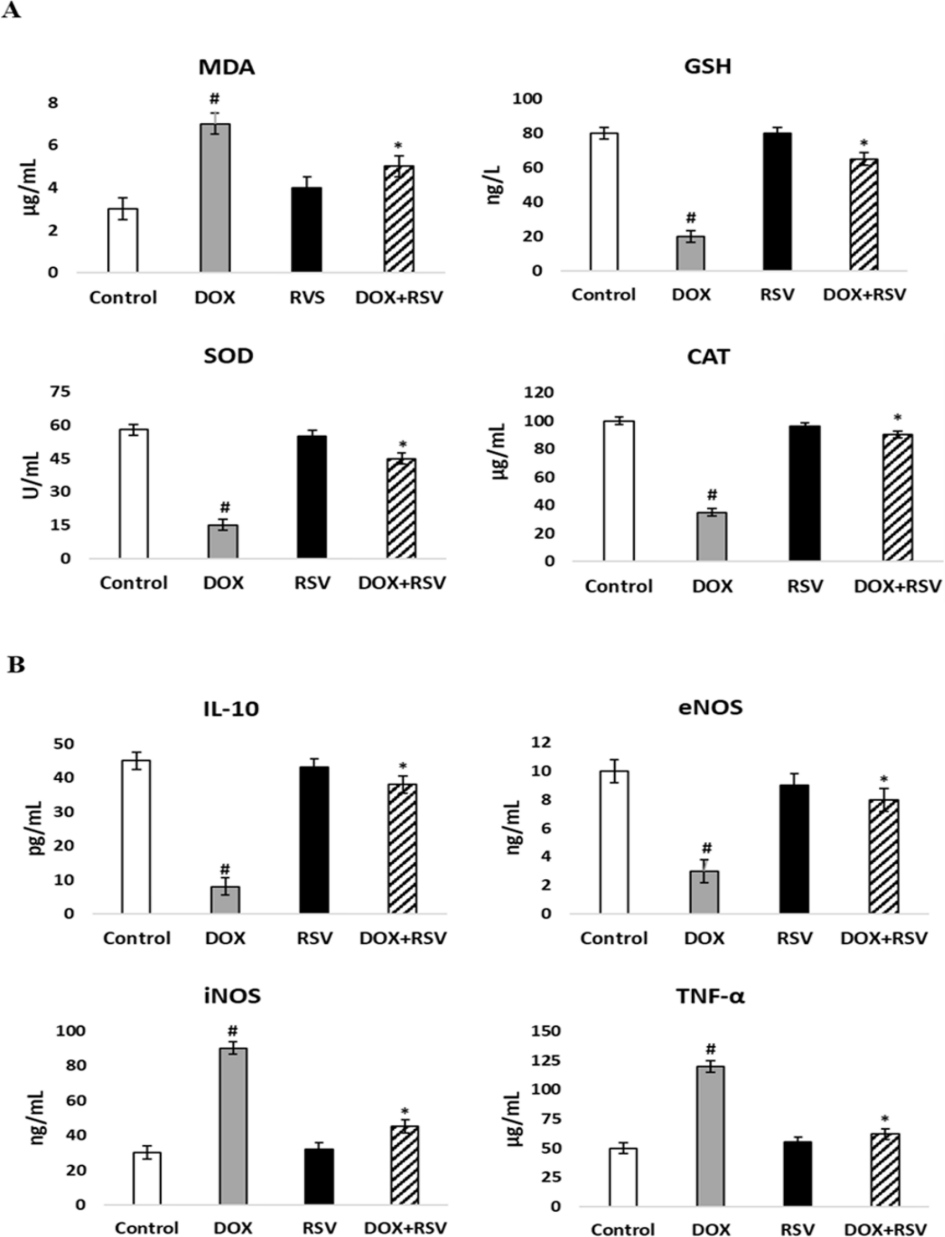
Effect of RVS on oxidative stress (**A**) and inflammatory (**B**) markers in DOX-injected rats. Data are stated as the means ± SD (*n* = 8). #*p* < 0.001 versus the control group, *p < 0.001 versus DOX group. DOX: Doxorubicin, RVS: 10 mg/kg Rosuvastatin, DOX + RVS: Doxorubicin + 10 mg/kg Rosuvastatin

In the present study, animals receiving DOX alone showed a statistically significant increase in MDA (7 ± 1.82 µg/mL, *p* < 0.001) compared to the control group. On the other hand, co-treatment with RSV revealed that animals showed a significant decrease in high MDA (5 ± 2.34 µg/mL, *p* < 0.001) levels. In addition, the DOX-only treated group showed statistically significant changes in antioxidant indices as indicated by decreased SOD (15 ± 1.25 U/mL, *p* < 0.001), GSH (20 ± 1.44 ng/L, *p* < 0.001), and CAT (35 ± 2.61 µg/mL, *p* < 0.001) enzymatic activities. However, the simultaneous administration of RSV in the treatment groups significantly reversed the increased SOD (45 ± 2.78 U/mL, *p* < 0.001), GSH (65 ± 3.21 ng/L, *p* < 0.001), and CAT (90 ± 6.18 µg/mL, *p* < 0.001) activities. No significant changes were observed in rats treated with RSV alone (Fig. 3A).

DOX alone administration resulted in a statistically significant decrease in IL-10 (8 ± 4.12 pg/mL, *p* < 0.001) and eNOS (3 ± 1.13 ng/L, *p* < 0.001) levels and a significant increase in iNOS (90 ± 5.96 ng/L, *p* < 0.001) and TNF-α (120 ± 18.02 µg/mL, *p* < 0.001) levels compared to the control group. In contrast, concurrent administration of RSV improved IL-10 (38 ± 7.56 pg/mL, *p* < 0.001), eNOS (8 ± 3.01 ng/L, *p* < 0.001), iNOS (45 ± 2.74 ng/L, *p* < 0.001) and TNF-α (62 ± 8.19 ug/mL, *p* < 0.001) levels in DOX-treated animals. No significant changes were observed in rats treated with RSV alone (Fig. 3B).

### RSV Alleviated DOX-Induced Cognitive Impairment by Activating the BDNF/CREB/ERK1/2 Pathway

**Fig. 4:**
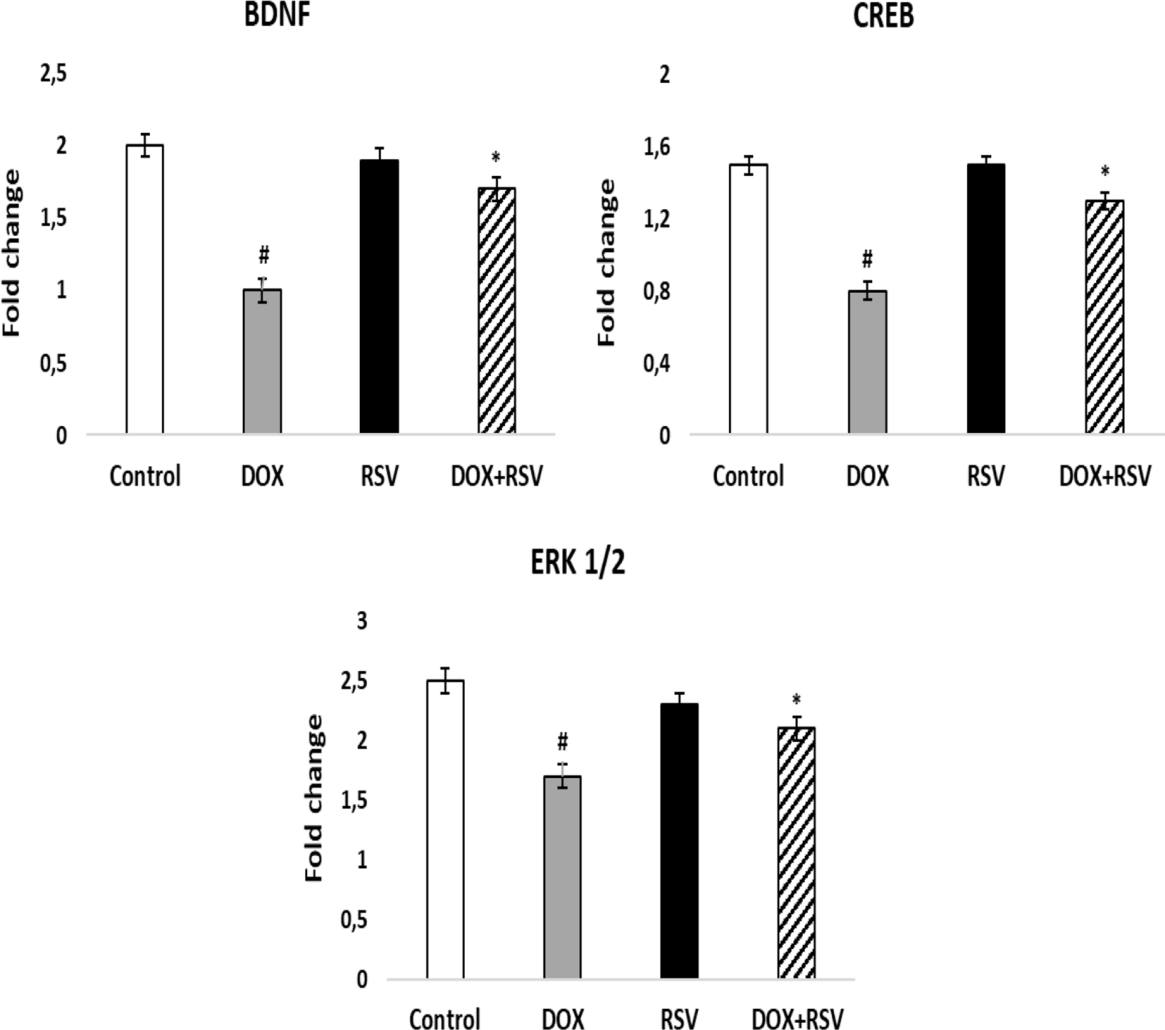
The effect of RVS on BDNF/CREB and ERK1/2 signaling in the brain. Data are stated as the means ± SD (*n* = 8). #*p* < 0.001 versus the control group, *p < 0.001 versus DOX group. DOX: Doxorubicin, RVS: 10 mg/kg Rosuvastatin, DOX + RVS: Doxorubicin + 10 mg/kg Rosuvastatin

We measured the levels of BDNF, CREB, and ERK1/2 to investigate the potential modulatory effect of RSV on synaptic plasticity. Gene expression results disclosed that DOX therapy significantly reduced BDNF, CREB, and ERK1/2 levels by 50%, 53%, and 113%, compared to the control group (*p* < 0.001). Conversely, RVS co-therapy indicated a significant rise in BDNF, CREB, and ERK1/2 levels of 85%, 86%, and 140%, respectively, compared with the DOX group (*p* < 0.001) (Fig. 4). In line with these findings, RVS increased synaptic growth factors by activating CREB, ERK 1/2, and BDNF in DOX- induced cognitive impairment.

### Histopathological Examination

**Fig. 5:**
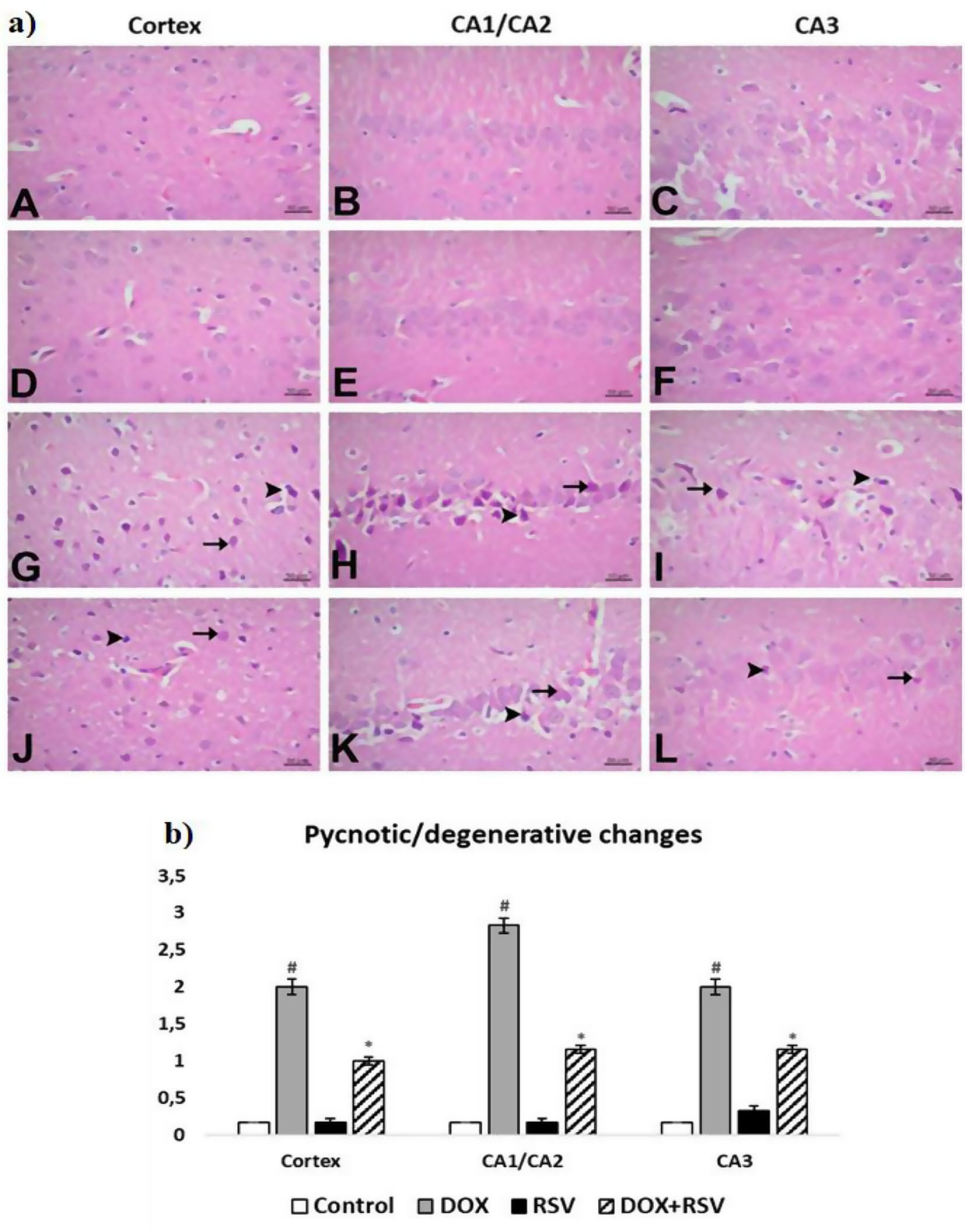
**a**) Histopathological images of experimental groups. Control group (**A**, **B**, **C**), RVS group (**D**, **E**, **F**). Normal histological appearance, DOX group (**G**, **H**, **I**). Pycnotic and degenerative neurons moderate in prefrontal cortex, severe in CA1/CA2 and moderate in CA3, DOX + RVS group (**J**, **K**, **L**). Mild pycnotic and degenerative neurons in the prefrontal cortex, CA1/CA2, and CA3. (►= pycnotic neuron, → =degenerative neuron), **H**-**E**. **b**) The number of pyknotic/degenerative changes is given. #*p* < 0.001 versus the control group, *p < 0.001 versus DOX group, (*n* = 8)

The control and RVS groups had a standard histological view. In the histopathological examination, pycnotic/degenerative alterations were watched in the prefrontal cortex, cornu ammonis parts of the brain. In the DOX group, pycnotic/degenerative alters were moderate in the cornu ammonis (CA3) and the prefrontal cortex parts and severe in the cornu ammonis (CA1/CA2) parts. Neurons in the prefrontal cortex and cornu ammonis were observed to be regularly distributed, with their nuclei becoming hyperchromatic and their cytoplasm shrinking, exhibiting a pyknotic-degenerative appearance. In the DOX + RVS group, these histopathological findings were found to be light. In neurons in the cornu ammonis and prefrontal cortex, pycnotic/degenerative findings were mild. While the nuclei in pycnotic neurons were dark and shrunken, it was determined that the structure of the nuclei began to deteriorate and became pale in degenerative neurons. Statistically significant distinctions were found between the groups in the histopathological evaluation (Fig. 5).

### Immunohistochemical Staining

**Fig. 6:**
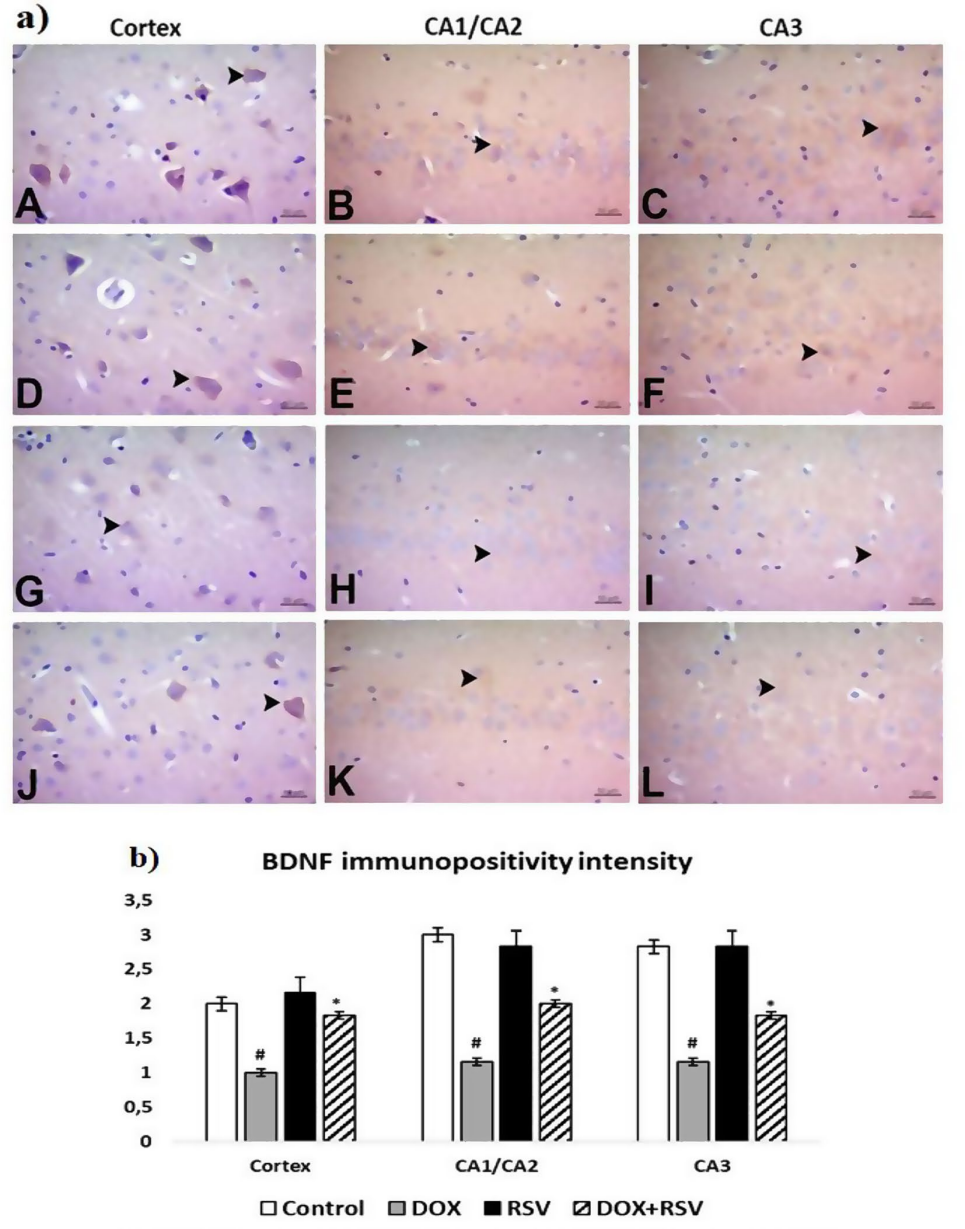
**a**) BDNF immunopositivity. Control group (**A**, **B**, **C**), RVS group (**D**, **E**, **F**). Moderate immunopositivity in the prefrontal cortex, CA1/CA2 and CA3 severe immunopositivity, DOX group (**G**, **H**, **I**). Mild immunopositivity in the prefrontal cortex, CA1/CA2 and CA3, DOX + RVS group (**J**, **K**, **L**). Moderate immunopositivity in the prefrontal cortex, CA1/CA2 and CA3 (►), IHC. **b**) The number of BDNF immunopositivity intensity is given. #*p* < 0.001 versus the control group, *p < 0.001 versus DOX group, (*n* = 8)

**Fig. 7:**
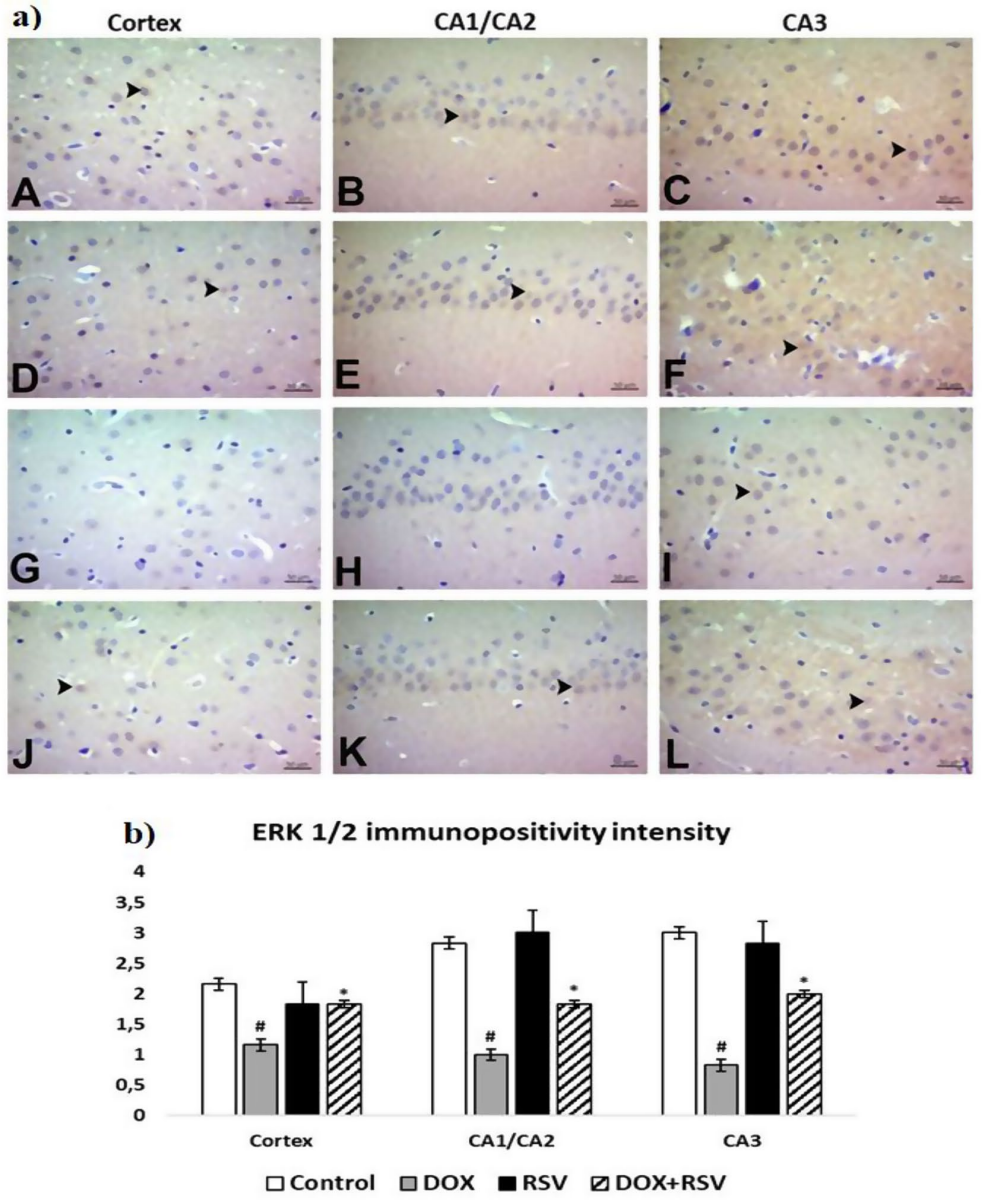
**a**) ERK 1/2 immunopositivity. Control group (**A**, **B**, **C**), RVS group (**D**, **E**, **F**). Moderate immunopositivity in the prefrontal cortex, CA1/CA2 and CA3 severe immunopositivity, DOX group (**G**, **H**, **I**). Mild immunopositivity in the prefrontal cortex, CA1/CA2 and CA3, DOX + RVS group (**J**, **K**, **L**). Moderate immunopositivity in the prefrontal cortex, CA1/CA2 and CA3 (►), IHC. **b**) The number of ERK 1/2 immunopositivity intensity is given. #*p* < 0.001 versus the control group, *p < 0.001 versus DOX group, (*n* = 8)

**Fig. 8:**
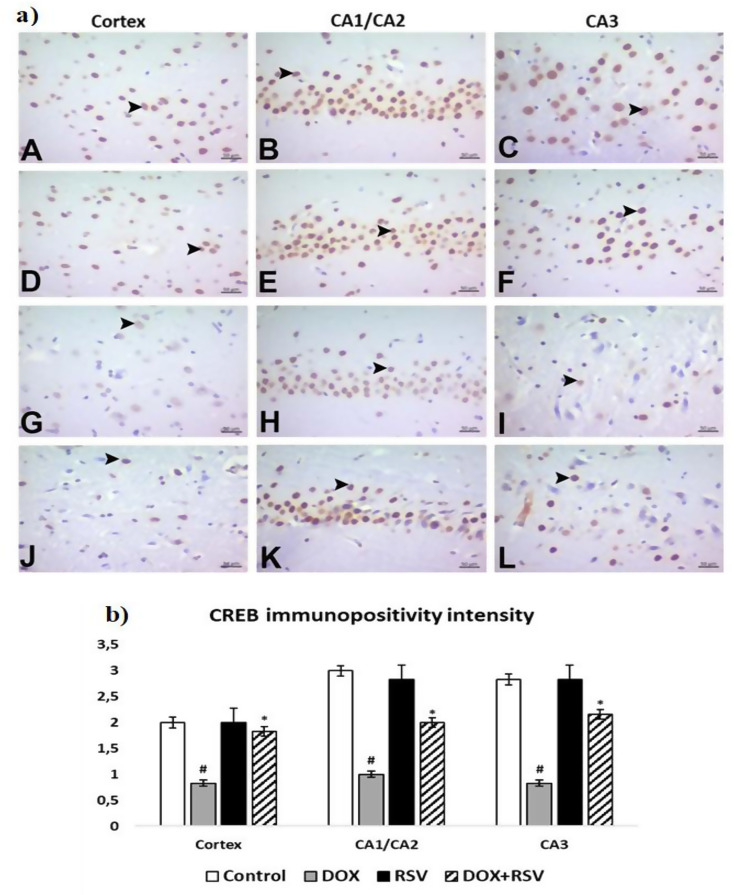
**a**) CREB immunopositivity. Control group (**A**, **B**, **C**), RVS group (**D**, **E**, **F**). Moderate immunopositivity in the prefrontal cortex, CA1/CA2 and CA3 immunopositivity in the prefrontal cortex, DOX group (**G**, **H**, **I**). Mild immunopositivity in the prefrontal cortex, CA1/CA2 and CA3, DOX + RVS group (**J**, **K**, **L**). Moderate immunopositivity in the prefrontal cortex, CA1/CA2 and CA3 (►), IHC. **b**) The number of CREB immunopositivity intensity is given. #*p* < 0.001 versus the control group, *p < 0.001 versus DOX group, (*n* = 8)

BDNF, ERK 1/2, and CREB immunopositivity were similar among themselves. In the control and RVS groups, moderate immunopositivity was found in the prefrontal cortex and severe immunopositivity in the cornu ammonis. In the DOX group, immunopositivity was light in the prefrontal cortex, cornu ammonis. In the DOX + RVS group, BDNF, ERK 1/2, and CREB immunopositivity were found to be moderate in the cornu ammonis and the prefrontal cortex. BDNF and ERK 1/2 were cytoplasmic, and CREB immunopositivity was nuclear. Statistically important distinctions were found between the groups in immunohistochemical painting for BDNF, ERK 1/2, and CREB (Figs. 6 and 7, and 8).

## Discussion

DOX is considered the most commonly used medicine in cancer therapy. Despite its clinical efficacy, DOX-derived cognitive impairment has received much attention as it severely impairs the quality of life of cancer survivors (Mani and Alshammeri 2023; Savran et al. 2024).

This work indicated that DOX administration significantly decreased step-by-step delay in the passive avoiding test and overall distance traveled in the locomotor activity test, in addition to cognitive impairment, as evidenced by the MWM analysis. In addition, the EPM analysis showed parallelism with these tests. These findings overlap with former works notifying DOX-induced cognitive disorder and fear conditioning in rats (Yu et al. 2022; Ali et al. 2022; Mani and Alshammeri 2023). In addition to neurobehavioral findings, histopathological examination showed severe nuclear pyknosis and neurodegeneration in hippocampal tissues of the DOX group. In contrast, co-therapy with RVS reduced these memory lacks in both histopathological and neurobehavioral experiments, which has previously proven the neuroprotective potency of RVS opposite neuroinflammation and memory disruption induced by a high salt and cholesterol diet (Husain et al. 2017).

Excessive free radical formation leads to oxidative stress, the primary mechanism responsible for the antineoplastic effect of DOX. In addition, since cell membranes are also affected by these radicals, it causes undesirable side effects (Mani et al. 2022). From this perspective, it has been suggested that oxidative damage plays an important role in DOX-induced cognitive impairment (Ebrahim et al. 2024). The cognitive impairment induced after DOX administration was observed to be associated with intense oxidative damage, as evidenced by decreased GSH, SOD, and CAT levels and increased MDA levels. Similarly, it has been previously shown that DOX-induced neurotoxicity is secondary to increased protein oxidation and lipid peroxidation, decreased GSH levels, in addition to impaired antioxidant enzyme activity (Savran et al. 2024). However, the co-administration of RVS significantly reduced DOX-induced oxidative stress. Relatedly, a double-blind, placebo-controlled, randomized clinical trial also investigated the effect of RSV on blood markers and the clinical status of mild AD patients with secondary dyslipidemia. The results of the study showed that it regulates oxidative stress markers (Rustamzadeh et al. 2024). Our current findings were consistent with previous studies reporting the protective effects of RVS against oxidative damage, potentially resulting from free radical scavenging activity by blocking the production and/or activity of reactive oxygen species or upregulation of cytoprotective enzymes. Further studies may focus on evaluating this effect with other oxidative/antioxidative markers (such as ischemia-modified albümin, total oxidant/antioxidant status, and thiol/disulfide homeostasis).

Oxidative stress and neuroinflammation are closely related to neurodegeneration. Circulating TNF-α crosses the blood-brain barrier and causes further oxidative and nitrosative damage to brain tissue. From the perspective of the nervous system, TNF-α is important in the development of neuronal damage because it also plays a role in the formation of neuroinflammation (Savran et al. 2024). A significant increase in plasma TNF-α levels was observed in cancer patients following treatment with chemotherapy protocols that included DOX (Tilija Pun and Jeong 2021). In line with this, systemic DOX administration produces inflammatory responses as evidenced by increased levels of TNF-α and iNOS in the hippocampus and cortex regions (Mounier et al. 2021; Mani and Alshammeri 2023; Lal et al. 2024). Our current results are consistent with previous results: IL-10, eNOS, iNOS, and TNF-α levels increased the neuronal toxicity of DOX. On the other hand, concurrent treatment with RSV suppressed DOX-induced neuronal toxicity. RSV-loaded human H-ferritin nanoparticles as brain-targeted nanoplatforms on secondary brain damage in intracerebral hemorrhage. Immunofluorescence staining results revealed that microglia and astrocytes were activated and largely infiltrated into hemorrhagic tissue in intracerebral hemorrhage mice, and RSV-loaded human H-ferritin nanoparticles significantly attenuated this uptake (Guo et al. 2025). These findings confirmed that RSV may have a neuroprotective effect via anti-inflammatory effects.

BDFN stated that dissimilar brain areas are responsible for maintaining synaptic plasticity and memory creation (Yang et al. 2022; Yu et al. 2022; Abdelaziz et al. 2025). In postmortem brain samples from depressed patients and mice exposed to chronic stress, BDNF mRNA and protein expression were found to be reduced, particularly in the hippocampus and amygdala (Yang et al. 2022; Xia et al. 2023). Moreover, BDNF specifically binds to the TrkB receptor, which may promote developmental neurogenesis and synaptic plasticity (Fang et al. 2024). The PI3K/Akt pathway is one of the cascades that regulate the functions of BDNF/TrkB signaling (Abdallah et al. 2021). Our results showed that RVS attenuated the DOX-related decrease in BDNF CREB, and ERK1/2 levels. CREB, a nuclear transcription factor, is responsible for forming new memories and regulates cognitive deterioration and neuronal survival (Yu et al. 2022; Abdelaziz et al. 2025). CREB can mediate the transcriptional activation of BDNF and serve as a target gene of CREB (Yu et al. 2022). According to the report, simvastatin administration can improve long-term potentiation induction dependent on PI3K/Akt signaling (Guo et al. 2021). Proof shows that simvastatin-mediated upregulation of BDNF and increased neurogenesis are beneficial for recovery from brain damage (Sultan et al. 2021). Simvastatin prohibits the disruption of neurogenesis from N-methyl-D-aspartate by rising BDNF in the brain (Yu et al. 2024). In addition, atorvastatin may promote functional recovery in stroke by increasing BDNF expression (Yu et al. 2022). Existing animal work on the effect of statins on neurotrophic factor levels has shown that they mediate the neuroprotective effect in neuropathological situations (Muneeb et al. 2022; Salari et al. 2024; Yu et al. 2024). In addition, ERK signaling has important functions in neurodegenerative and neuroinflammatory conditions (Stekic et al. 2022). DOX neurotoxicity is associated with excessive activation of ERK and PI3K/Akt regulation (Alhowail et al. 2023). Therefore, part of the underlying mechanism of RSV protection against DOX toxicity appears to be mediated through the modulation of PI3K/Akt-ERK. We propose that RSV alleviates DOX-induced cognitive impairment in adult rats by promoting BDNF release, which involves activation of the CREB-ERK pathway, by suppressing oxidative stress and inflammation. To our knowledge, this is the first study to provide evidence for the neuroprotective effect of RSV co-treatment in DOX-induced neurodegeneration.

The current study had some limitations. Oxidative stress and inflammation markers in brain tissue were not examined. This prevented us from directly explaining the relationship with cognitive impairment. Another limitation is the single application of DOX and RSV. Although neurotoxicity was proven by behavioral, biochemical, molecular, histological, and immunohistochemistry analyses in our model, comparative dose studies are needed. These parameters need to be evaluated in further dosing studies. Also, further mechanistic studies are needed to fully understand the effect of DOX and RSV on other brain regions. Additionally, RSV-induced neuroprotection can be addressed in female rats to investigate the gender effect.

## Conclusion

In conclusion, we defined that DOX application increased oxidative stress load and neurodegeneration following neuroinflammation in hippocampal and prefrontal cortex regions. An HMG-CoA reductase inhibitor, RVS, significantly reduces DOX-related oxidative stress, neuroinflammation, and neurodegeneration in the prefrontal cortex and hippocampus of adult rats. RVS exerts a neuroprotective effect by inducing BDNF expression, which includes activation of the CREB-ERK path. It suggests that RSV may help prevent or alleviate cognitive dysfunction in patients with various types of cancer undergoing chemotherapy due to BDNF/CREB/ERK 1/2 modulation and may have a potential benefit in neurodegenerative diseases.

## Data Availability

‘’The data supporting this study’s findings are available from the corresponding author upon reasonable request’’.
